# Unveiling the potential of phytochemicals to inhibit nuclear receptor binding SET domain protein 2 for cancer: Pharmacophore screening, molecular docking, ADME properties, and molecular dynamics simulation investigations

**DOI:** 10.1371/journal.pone.0308913

**Published:** 2024-08-20

**Authors:** Gamal A. Mohamed, Hossam M. Abdallah, Ikhlas A. Sindi, Sabrin R. M. Ibrahim, Abdulrahim A. Alzain

**Affiliations:** 1 Department of Natural Products and Alternative Medicine, Faculty of Pharmacy, King Abdulaziz University, Jeddah, Saudi Arabia; 2 Department of Biology, Faculty of Science, King Abdulaziz University, Jeddah, Saudi Arabia; 3 Department of Chemistry, Preparatory Year Program, Batterjee Medical College, Jeddah, Saudi Arabia; 4 Department of Pharmaceutical Chemistry, Faculty of Pharmacy, University of Gezira, Wad Madani, Sudan; Saveetha University - Poonamallee Campus: SIMATS Deemed University, INDIA

## Abstract

Nuclear receptor binding SET domain protein 2 (NSD2) significantly contributes to the development of cancer, making it a promising target for cancer drug discovery. This research explores natural compounds as potential selective inhibitors for NSD2 in cancer treatment. Employing a comprehensive *in silico* approach, the study utilized pharmacophore modeling, molecular docking, pharmacokinetic profiling, and molecular dynamics simulations. An e-pharmacophore model-based screening using the first selective and potent ligand bound to NSD2 identified 49,248 natural compounds from the SuperNatural 3.0 database (containing 449,008 molecules) with acceptable alignment with the developed pharmacophore hypotheses. Subsequently, molecular docking was executed to assess the standout compounds which led to the selection of ten candidates that surpassed the reference inhibitor in accordance w the binding affinity expressed as a G score. Ligand-residue interaction analyses of the top three hits (SN0450102, SN0410255, and SN0142336) revealed diverse crucial interactions with the NSD2 active site, including hydrogen bonds, pi-pi stacking, and hydrophobic contacts with key amino acid residues in the NSD2-PWWP1 domain. Pharmacokinetic profiling confirmed the drug-likability for the refined hits, indicating good cellular permeability and minimal blood-brain barrier penetration. Molecular dynamics simulations for 200 nanoseconds affirmed the stability of protein-ligand complexes, with minimal fluctuations in root mean square deviation and root mean square fluctuation analyses. Overall, this study identified promising natural compounds as potential pharmaceutical agents in the treatment of NSD2-associated cancers.

## Introduction

Cancer is a major global health concern, with millions of new cases and deaths reported annually. In 2018, there were approximately 18.1 M newly diagnosed cases in addition to 9.6 M cancer-related deaths worldwide [1]. These numbers are anticipated to repeatedly increase, to culminate in an annual count of nearly 22 million new cases by 2030 [2, 3]. Both genetic and epigenetic alterations contribute to the initiation and progression of cancers. Epigenetic aberrations, unlike genetic alterations, can be reversed, allowing malignant cells to return to a lesser aggressive state. Epigenetic therapy i.e., targeting critical regulators of gene expression and chromatin integrity that are dysregulated in various human cancers, has emerged as an effective approach for both chemotherapy and chemoprevention of cancer [4].

The nuclear receptor-binding SET domain (NSD), a family of histone lysine methyltransferases, including NSD1, NSD2/WHSC1/MMSET, and NSD3/WHSC1L1, have been identified as potential therapeutic targets for cancer [5]. These enzymes perform vital functions in chromatin regulation and have been implicated in various cancers [6–10]. NSDs activation, including NSD1, NSD2, and NSD3 has been found to be tightly associated with the occurrence and progression of different cancer types [8]. Beside cancers, NSD2 is also linked with other diseases, such as inflammatory and autoimmune diseases. In this research, we focus in discovering NSD2 inhibitors for cancer, as it is the most directly related and extensively investigated disease linked with NSD2 abnormalities compared to other diseases.

NSD2, specifically, regulates chromatin through methylations of H3K36 and H4K20, although it also shows substrate specificity for other histone marks (H3K4, H3K9, H3K27, H3K79, and H4K20), indicating a complex and not fully understood regulatory mechanism [6, 11, 12]. NSD2 overexpression enhances cellular proliferation, affects chromosomal accessibility, and alters gene expression regulation [11]. It is involved in DNA damage repair, being recruited to double-strand breaks, and increasing H4K20 methylation levels at these sites [12]. The short isoform of NSD2, REIIBP, increases H3K27 methylation and grabs histone deacetylases [13]. REIIBP can additionally methylate H3K79, which was previously assumed to be exclusively methylated by DOT1L [14].

NSD2 overexpression is linked to the aggressiveness of tumors and is involved in over 20 types of cancer [14, 15]. It is frequently mutated in pediatric cancers and mantle cell lymphoma and it is highly expressed in various other cancer types [16–20]. NSD2 overexpression is a characteristic feature of the translocation in multiple myeloma. This translocation is linked with a poor prognosis for patients. Multiple myeloma itself accounts for 20% of deaths related to hematological malignancies which is currently with no cure [21]. Targeting NSD2, especially in multiple myeloma, holds potential for cancer therapy [15, 22]. Despite considering NSD2 as a promising target in cancer therapy, the development of selective small molecule inhibitors for NSD2 has been challenging [23, 24]. While some inhibitors have shown activity against NSD2, their selectivity against other NSD enzymes (NSD1 and NSD3) is uncertain [25, 26]. Existing inhibitors, such as MCTP39, Sinefungin, and PTD2, demonstrate inhibitory effects on NSD2 but lack selectivity for NSD1 and NSD3 [22, 27]. Inhibitors targeting G9a/G9a-like protein (GLP) H3K9 HMTase, like BIX-01294 and BIX-01338, also exhibit inhibitory activity against NSD1, NSD2, and NSD3 [28]. KTX-1001 as a new inhibitor NSD2 for the SET catalytic site, was advanced to a phase I clinical trial for the treatment of patients with recurrent and refractory MM. There is no detailed information on KTX-1001, including its structure and preclinical data [29]. Taken together, the development of selective inhibitors for each NSD enzyme, particularly NSD2, has yet to be accomplished and is urgently needed [30, 31].

The scientific literature demonstrates a strong interest in screening natural compounds for the development of medications, particularly in the field of cancer treatment [32]. Over 60% of current anticancer drugs are derived from natural sources or their derivatives [33]. Natural compounds are favored for their affordability, minimal side effects, and high tolerability by the body, making them potentially effective against medication resistance in cancer [34].

Advancements in technology have revolutionized the application of computational strategies in drug development. Currently, these approaches are frequently used at several phases of the drug development process, including hit recognition, lead optimization, and formulation design [35–41]. In the specific context of developing NSD2 inhibitors, a ligand-based virtual screening study was conducted using various computational techniques. The study employed a combination of computational methods to find fundamentally new NSD2 inhibitors including, pharmacophore modeling, molecular docking, and MD simulations.

## Methods

All computational studies were carried out using maestro v 12.8 of Schrodinger and the academic Desmond v6.5 by D.E. Shaw Research for molecular dynamics.

### Preparation of protein

The X-ray crystallographic structure of the NSD2 protein (PDB ID: 6G2O) was obtained from the open-access Protein Data Bank website and prepared using the protein preparation wizard provided by Schrödinger software [42]. The PDB file initially contains heavy atoms and may include water molecules, cofactors, activators, ligands, metal ions, and protein subunits. However, it lacks assigned ionization and tautomeric states, and there may be missing side chains. Additionally, information about bond order and atomic charges is absent. To address these structural issues, the protein preparation wizard was utilized. The protein structure was minimized using the OPLS4 force field, and various modifications were made, including adding hydrogen atoms, fixing residue charges, assigning bond orders, and creating disulfide bonds [43]. The flip-states of histidine residues were also adjusted. In the final step, the entire structure underwent energy minimization using the impref utility, which employs minimization cycles based on the OPLS4 force field and the impact molecular mechanics engine. The restrained minimization process continued until the RMSD value of heavy atoms reached a threshold of 0.3 Å relative to the starting X-ray structure. The resulting modified protein structure, obtained after the preparation steps, was then used for the computational studies.

### E-pharmacophore generation

Structure-based pharmacophore design is an approach used to create a pharmacophore model based on the structural characteristics of the protein target and the bioactive conformation of a ligand that is co-crystallized with the protein. This method relies on the analysis of the stable active site of the protein target and the specific conformation and binding interactions observed in the X-ray crystallographic structure of the ligand-protein complex [44]. The pharmacophore modeling in this study was performed using the PHASE module of the Schrödinger suite [45]. The PHASE module generates pharmacophore hypotheses based on the features observed in the co-crystallized ligand. The pharmacophore skeleton utilized in this study consisted of six features: HB-donor (D), HB-acceptor (A), hydrophobic region (H), negatively charged region (N), positively charged region (P), and aromatic rings (R) [46]. The generated pharmacophore hypotheses were then ranked using scoring parameters and aligned against the co-crystallized ligand. This ranking process helps identify the most relevant pharmacophore hypotheses. Finally, the generated pharmacophore hypothesis was applied to a database to retrieve hits with higher potency than the reference ligand.

### Screening of the SN.3 database

SN.3 natural compound database, consisting of approximately 449,008 compounds, was screened to hunt potential inhibitors for the NSD2 receptor. The compounds in the database were first minimized using the MacroModel module available in the Schrödinger suite. This minimization process ensures optimized molecular structures and conformations of the natural compounds. Following that, the minimized NPs database was screened utilizing the PHASE module’s sophisticated pharmacophore-screening function, based on the verified pharmacophore hypotheses. The screening process aims to find compounds that match the specified pharmacophore features and exhibit potential inhibitory activity against the NSD2 receptor. In this screening step, four out of the four pharmacophore features were designated as "must match," meaning that compounds must possess these features to be considered as hits.

### Molecular docking

Molecular docking is a prevalent computational approach for predicting the affinity of binding for the protein and ligand complexes. In this study, the Glide module of the Schrödinger Suite was utilized for molecular docking to identify potential "hit" molecules obtained from the pharmacophore-based screening [47]. The prepared NSD2 protein structure served as the receptor for the docking process. A receptor grid was generated based on the conformation of the co-crystal ligand, which helped in the definition of the active amino acids within the grid for accurate docking [47]. The docking was done in a step-by-step manner using two modes: HTVS and XP. HTVS is a fast-screening process used for the preliminary docking of large databases. The top 100 HTVS scoring ligands were chosen and underwent XP docking, which provides more detailed and accurate results by considering precise interaction modes between the ligand and the protein. The docking parameters and settings involved adding the receptor grid file to the receptor grid box and the prepared ligands to the ligands tab. The first docking stage used HTVS, followed by XP precision in the subsequent stage [48]. Default parameters were employed for other parameters. The docking outcomes were analyzed and tabulated based on the G-scores, which indicate the binding affinity and represent the quality of the protein-ligand interactions.

### Pharmacokinetic profile prediction

QikProp, a module within the Maestro program, is a tool used to calculate unique ADME-relevant descriptors [49]. It offers a novel approach for optimizing the pharmacokinetic profile of pharmaceutical compounds. Recognizing the importance of favorable pharmacokinetic features in successful drug discovery, ADME assessments have been integrated earlier into drug design strategies. In this study, QikProp was employed to establish correlations between 3D molecular structures and physicochemical and pharmacokinetic properties. The top 3 molecules were subjected to energy minimization to obtain their minimum energy conformations. ADME properties were then calculated based on these conformations.

### Molecular dynamics

To refine the NSD2 receptor complexes with the top three lead molecules and the co-crystal ligand, MDs were performed using the Desmond software [50–52]. The consistency of their interactions was studied through these simulations. The process began by using the system builder application of the Desmond module. Default parameters were used, then the system was solvated with a TIP3P water model in an orthorhombic periodic boundary box. The box buffer size dimensions were set to 10 Å in each direction (a:10 × b:10 × c:10). To neutralize the system, Cl− ions were added based on the total charge of the model, along with a salt concentration of 0.15 M. The next step involved minimizing the model obtained from the system builder. The minimization application was used, with the maximum iterations set to 2000, and the remaining parameters were kept at their default values. The then system was subjected to the MD production phase. This phase of the MD simulation is divided into seven different stages as follow:

stage 1 –task.

stage 2—simulate, Brownian Dynamics NVT, T = 10 K, small timesteps, and restraints on solute heavy atoms, 100ps.

stage 3—simulate, NVT, T = 10 K, small timesteps, and restraints on solute heavy atoms, 12ps.

stage 4—simulate, NPT, T = 10 K, and restraints on solute heavy atoms, 12ps.

stage 5—simulate, NPT and restraints on solute heavy atoms, 12ps.

stage 6—simulate, NPT and no restraints, 24ps.

stage 7 –simulate.

The first six stages include the equilibrium phase and consist of short simulation steps. Step 7 is a last, long simulation stage. A total of 200 ns production stage was carried out.

## Results and discussion

**Fig 1:**
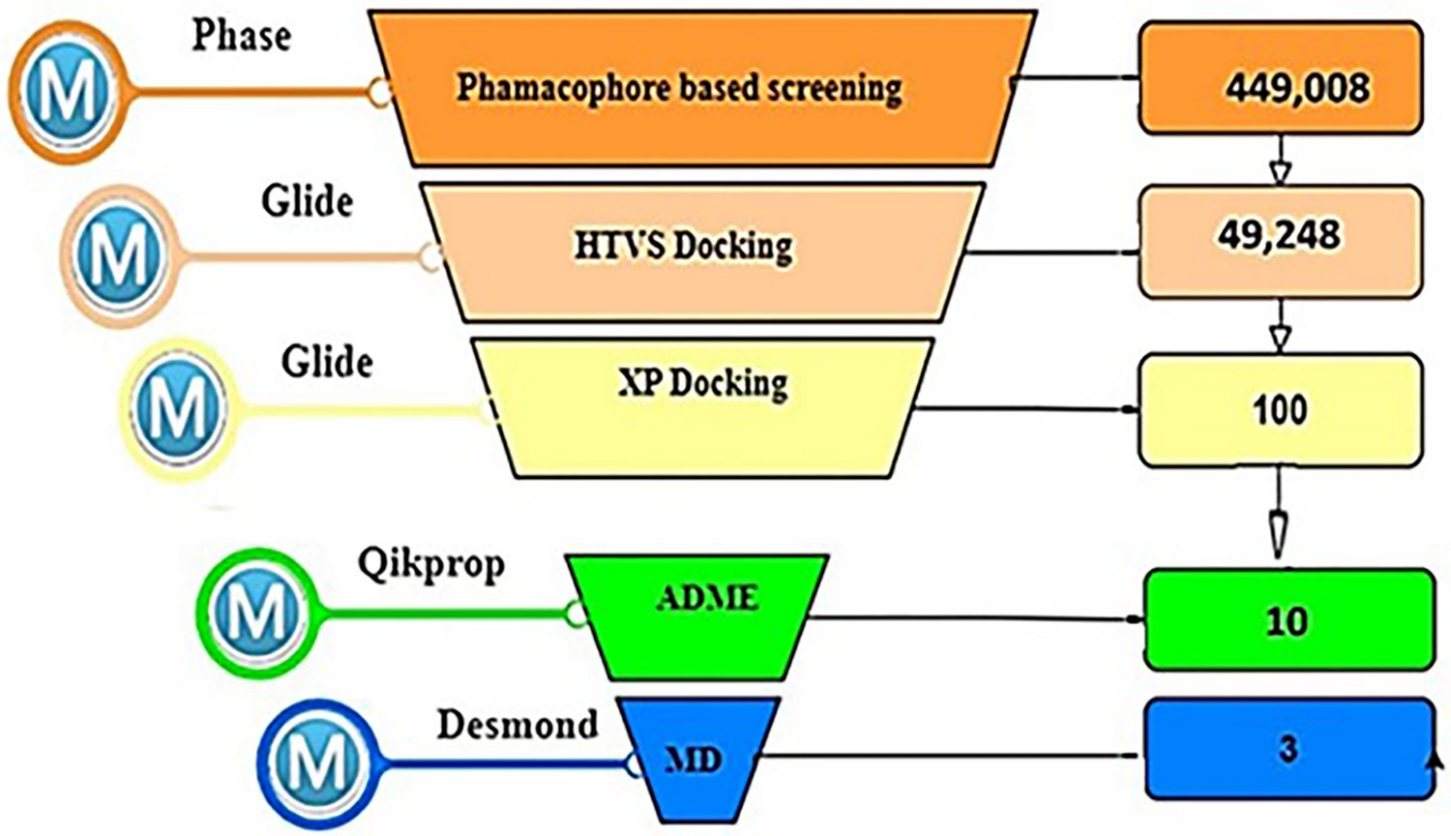
The overall study workflow.

The workflow of this study is summarized in **Fig 1**.

### Pharmacophore modeling

**Fig 2:**
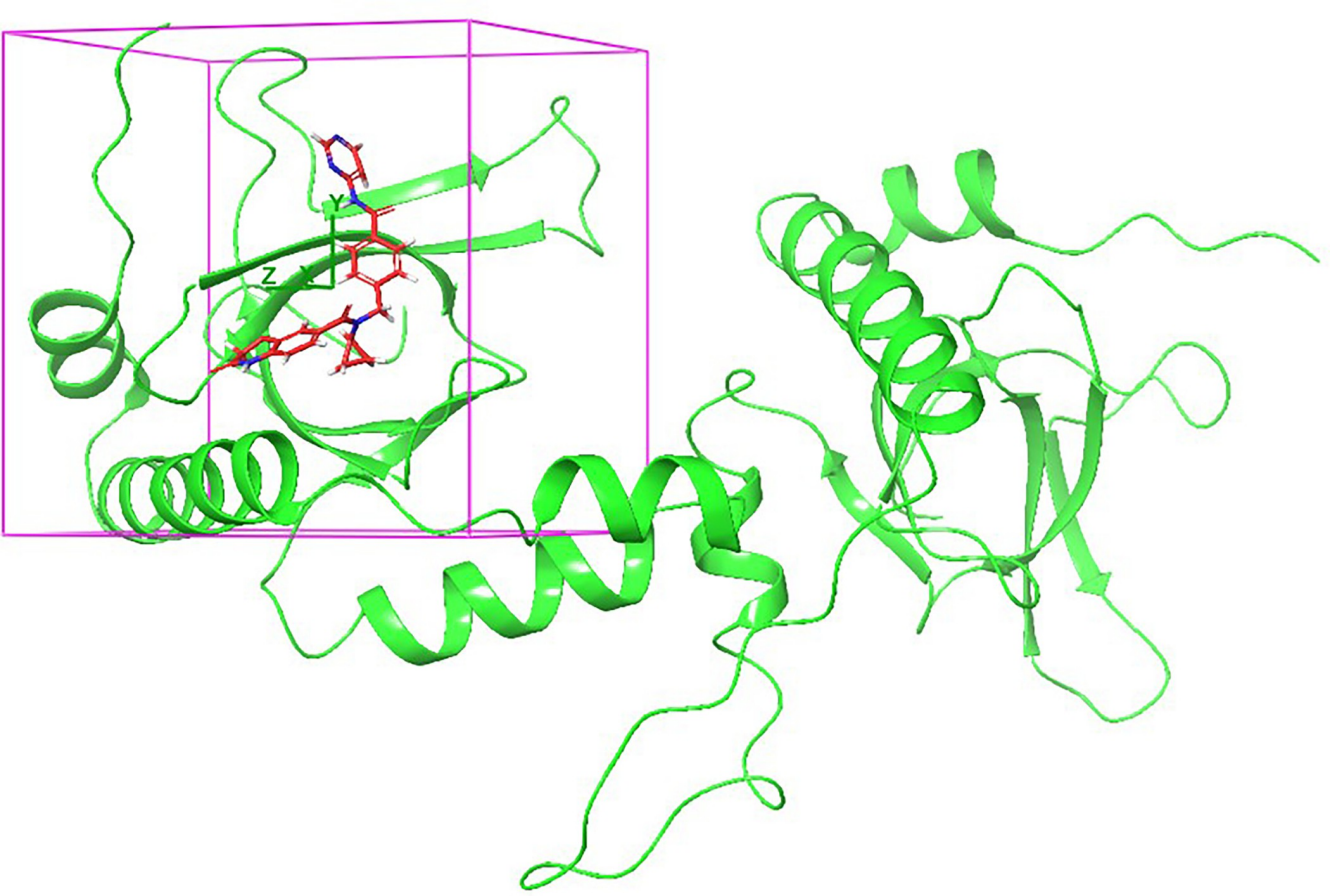
The structure of NSD2 bound to the co-crystallized ligand in the active site (pink box) (PDB ID: 6G2O).

**Fig 3:**
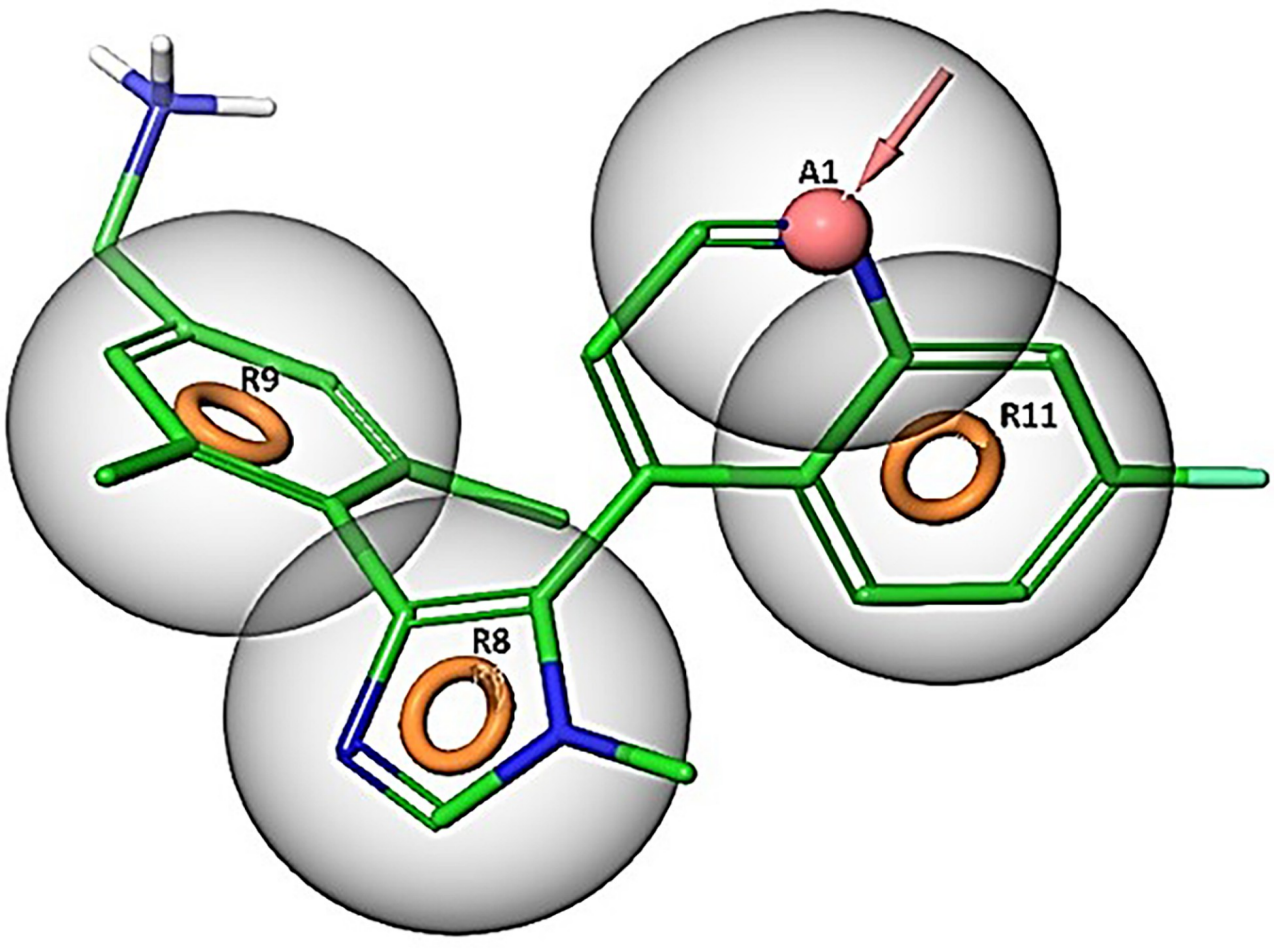
The generated pharmacophore hypothesis based on the co-crystallized ligand of the active site domain (PDB ID: 6G2O). (R8), (R9), (R11) doughnut-shaped circles denoting the aromatic ring structures and A1 denoting for the hydrogen bond acceptor.

Depend on the targeted domain, NSD2 small molecules inhibitors are classified into three classes: catalytic SET domain of NSD2 (NSD2-SET) inhibitors, PHD domain of NSD2 (NSD2-PHD) inhibitors, and PWWP1 domain of NSD2 (NSD2-PWWP1) inhibitors. Drug discovery projects targeting the catalytic SET domain yielded little success. PPI domains, including PHD domains and PWWP domains (PWWP1 and PWWP2) could be clinically relevant and selectively blocked by compounds [29]. In 2021, to identify a selective NSD2 inhibitors targeting the NSD2-PWWP1 domain, molecular docking and experimental validation were performed [29]. A new scaffold was identified and provided the basis for further SAR studies. This research employed NSD2 structure for the generation of an e-pharmacophore model with a specific focus on the PWWP1 domain. The pharmacophore model was created based on the distinctive structural properties observed in the co-crystallized inhibitor **(Fig 2)**. Consequently, this led to the identification of four pharmacophoric features (three-ring structures and a hydrogen bond acceptor). Accordingly, a library containing 449,008 natural compounds retrieved from the SN.3 database was screened against the generated pharmacophoric features. As a result, a total of 49,248 candidate molecules that possessed three-ring structures in addition to a hydrogen-accepting moiety have been filtered in as matching the pharmacophore hypotheses. Subsequently, to validate the results of the match, molecular docking was performed on the active site of the NSD2 domain. **Fig 3** depicts the pharmacophoric features of the PWW1 binding domain.

### Molecular docking analysis

Molecular docking is an integral part of computational drug discovery. Basically, it is introduced to study the interactions between a target entity and a binder in terms of bonding and non-bonding interactions. While molecular docking has a limitation of not accounting for the flexibility of receptors, it continues to be a valid method for providing initial insights [53, 54].

In Glide, the HTVS procedure was done on 49,248 compounds that matched the pharmacophore hypotheses. Afterward, the top 100 molecules of the pool were taken for subsequent extra-precision (XP) docking (docking scores: -9.061 to -7.164 kcal/mol, S1 Fig in S1 File), since the remaining molecules showed low docking scores < -6.00 kcal/mol. The XP mode docking is recognized for its increased accuracy and precision compared to other modes. This mode was employed for the initial screening of the compounds and consequent filtering that relies on their XP docking scores. The scoring function integrated into Glide combines empirical and force-field-based factors to compute binding energy, facilitating the filtration of the most optimal docking poses [47, 55]. In reference to Table 1, it can be clearly noticed that there are 10 compounds having docking scores better than that of the co-crystallized reference inhibitor. In this regard, the quantitative assessment of the binding is taken via docking score. The docking score reflects the binding affinity of the docked entities. Whereby, the more negative the docking score, the better the binding affinity and the merrier binding stability.

**Table 1:** The docking scores of the top 10 ranked hits and the co-crystallized reference inhibitor.

Compound ID	Docking score (kcal/mol)
SN0450102	-12.041
SN0410255	-10.620
SN0142336	-9.944
SN0016799	-9.757
SN0038102	-9.753
SN0383088	-9.740
SN0232449	-9.688
SN0030463	-9.642
SN0432732	-9.604
SN0346214	-9.519
NSD2 reference	-9.355

The co-crystallized inhibitor was identified as the first potent and selective NSD3-PWWP1 antagonist (IC_50_ = 0.2 μM), which can disrupt the interactions between NSD3-PWWP1 and H3K36me2, leading to a significant reduction of MYC mRNA levels and suppressing the proliferation of leukemia cell lines [56]. As in Table 1, it can be seen that this potent inhibitor presented a docking score of -9.355 kcal/mol. This in turn stands as a reference point for the docked library of compounds. Ten out of the top hundred molecules showed outstanding scores of dockings that surpass that of the potent inhibitors. The docking scores of the ten compounds ranged from -12.041 kcal/mol to -9.519 kcal/mol. As long as these top-ranked hits have superior binding affinities, they are anticipated to possess a better activity in further experiments. However, a more detailed explanation of their precise binding mechanism is warranted.

### Ligand-residue interaction pattern analysis

**Fig 4:**
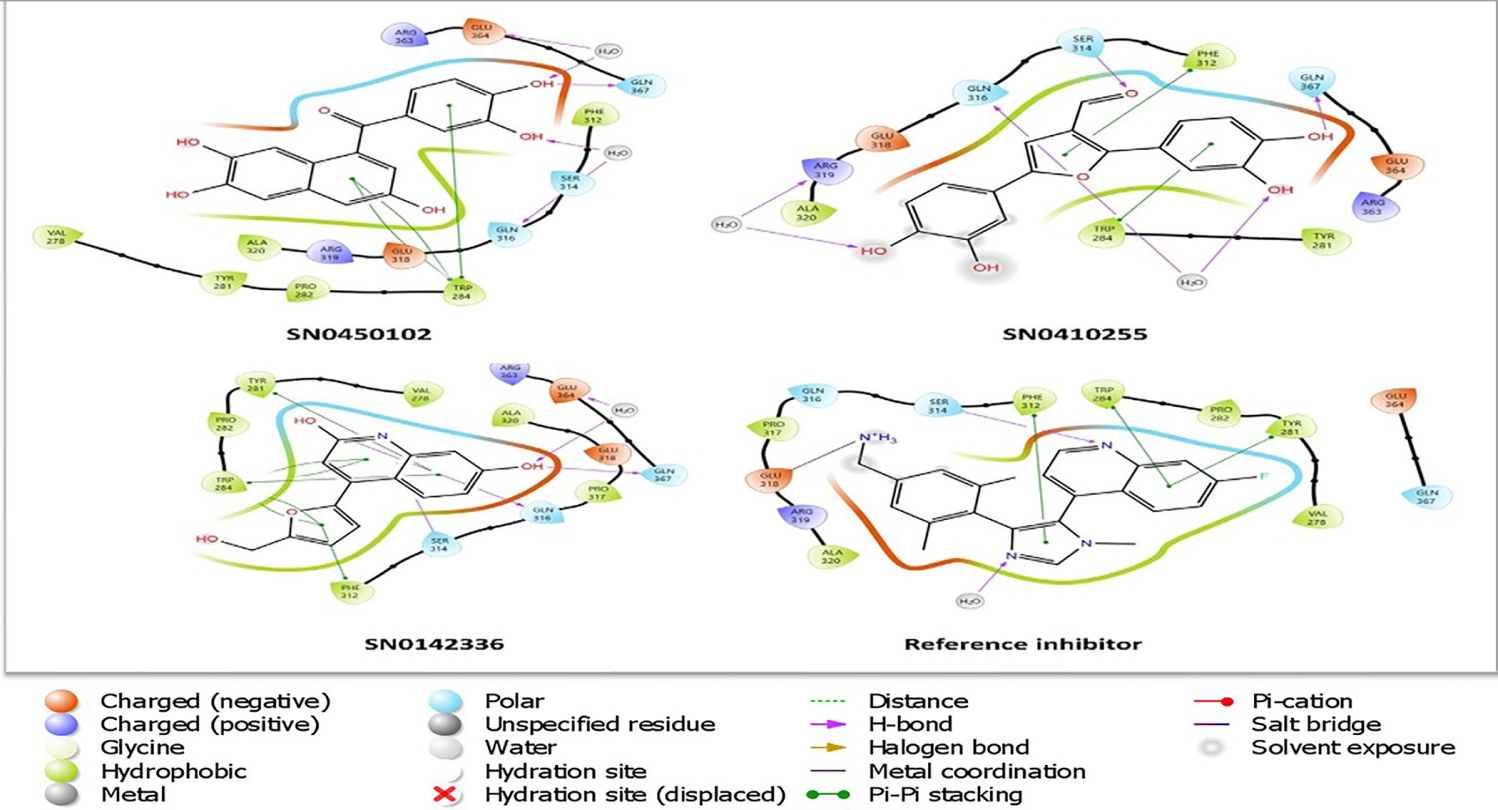
The two-dimensional interactions of the top-docked inhibitors and the co-crystallized reference inhibitor on NSD2 active site domain (PDB ID: 6G2O).

**Fig 5:**
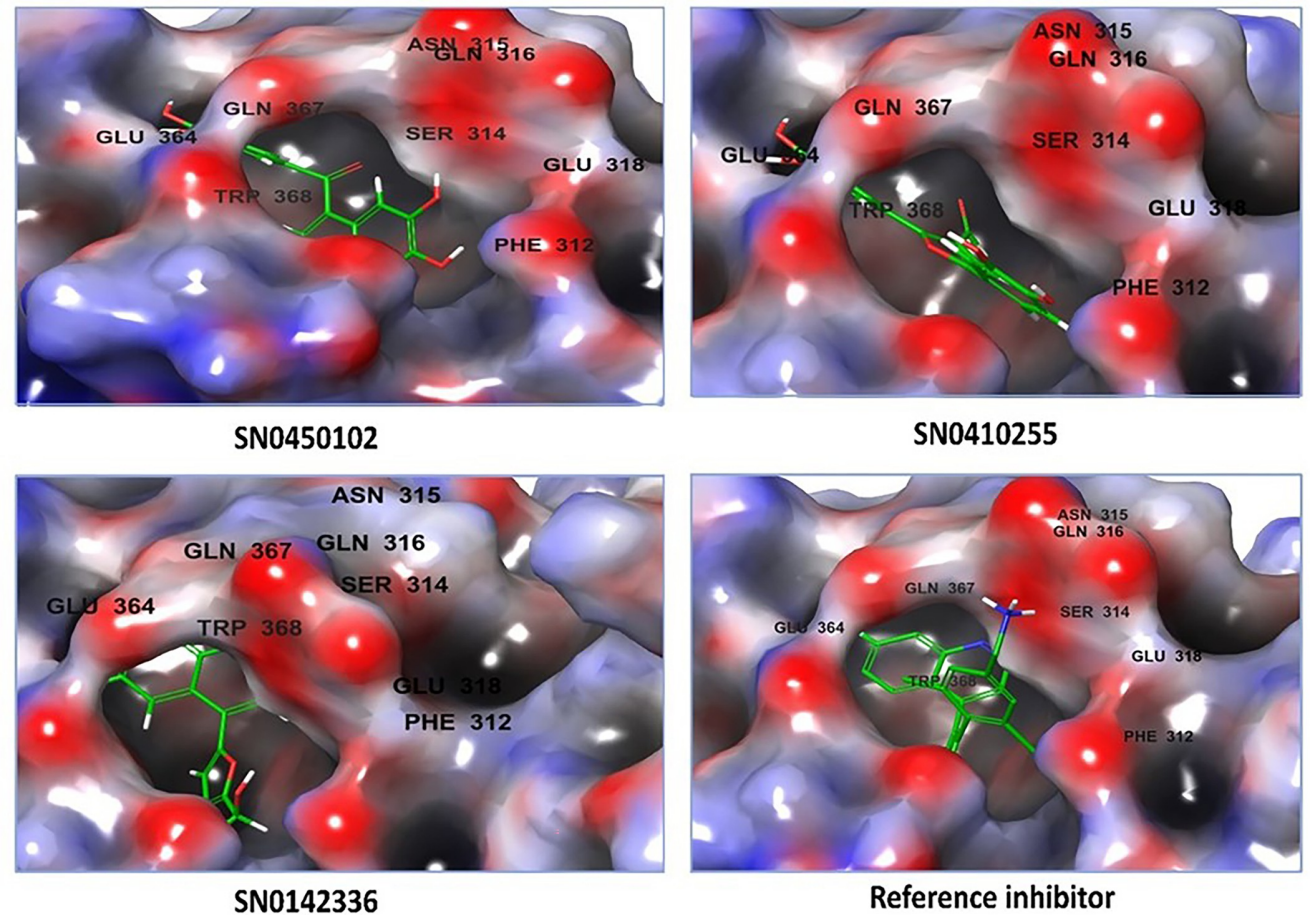
The three-dimensional interactions of the top-docked inhibitors and the co-crystallized reference inhibitor on NSD2 active site domain (PDB ID: 6G2O).

According to the previously reported work of Ma *et al*. [29], in the crystal structure of the active domain of NSD2-PWWP1 (PDB ID: 6G2O), the recognized key amino acid residues are SER-314 and GLU-318. Encouragingly, the co-crystallized inhibitor that was docked in the active site domain reproduced the same reported interactions with the aforementioned key amino acid residues. This is consistent with the previously reported binding mode [29]. For the purpose of taking the compounds for further analysis, the top-scoring three compounds will be discussed and taken as representatives due to the high computational cost of handling a large number of compounds. The focus on the details of the interacting amino acid residues and their corresponding parts in contact with the ligands demonstrates the nature of the interactions. To attain this, the ligand interactions diagram panel of the Maestro interface was utilized to display the two-dimensional interactions in Fig 4. Likewise, Fig 5 depicts the three-dimensional interactions of the docked compounds on the active site pocket showing the electrostatic potential map.

According to Fig 3, the co-crystallized reference inhibitor showed the ability to constitute direct hydrogen bonds to SER-314 (1.95 Å), the key amino acid of NSD2, and a slat bridge with the other key amino acid GLU-318 (3.26 Å). Addedly, a form of hydrophobic interaction known as pi-pi stacking was also presented between the ring structures of the reference compound and the amino acid residues TRP-284, TYR-281, and PHE-312.

The first hit, SN0450102 which is extracted from the plant *Curculigo sinensis* [57] and is chemically known as 8-(3,4-dihydroxybenzoyl)naphthalene-2,3,6-triol showcased interactions of different types. Particularly, it presented a direct hydrogen bond to the amino acid residue SER-367 (1.88 Å) together with indirect water-mediated hydrogen bonds to GLU-364 (3.93 Å) and GLN-316 (4.17 Å). Additionally, it can be seen that pi-pi stackings are also occurring with TRP-284.

The second hit, SN0410255 is reported to be produced by *Molineria crassifolia* and is chemically identified as 2,5-bis(3,4-dihydroxyphenyl)furan-3-carbaldehyde [57]. This molecule also exhibited a direct hydrogen bond to the essential amino acid residue SER-314 (2.14 Å) and another hydrogen bond to GLN-367 (1.8 Å). Water-mediated hydrogen bonds were also prevalent with GLN-316 (4 Å) and ARG-319 (3.56 Å). The pi-pi stacking was apparent with TRP-284 and PHE-312 as a sort of interaction that strengthened the binding to the active pocket.

The third hit, SN0142336, an extract of the plant *Aquilegia ecalcarata* [58] that is chemically recognized as 7-hydroxy-4-[5-(hydroxymethyl)furan-2-yl]-1,2-dihydroquinolin-2-one, constituted a direct hydrogen bonding to the key amino acid residue SER-314 (1.96 Å). Two more hydrogen bonds to GLN-367 (1.7 Å) and GLN-316 (1.59 Å) were also noticed. In addition, water-bridged hydrogen bonds were also seen with GLU-364 (4.4 Å). Besides, multiple pi-pi stackings with TYR-281, TRP-284, and PHE-312 were also seen.

In summary, as the docking process is known to provide meaningful glimpses into the binding mode, the affinity of binding, and the receptor-ligand interaction pattern, the docking results of these three hits disclosed evaluable information about their binding affinity and how they behave with respect to the constitutional amino acid residues inside NSD2 receptor cavity.

### Pharmacokinetic profiling

Prior to subjecting the highest-ranked hits to additional computational evaluation, it was imperative to address the critical issue of pharmacokinetic profiling. Consequently, the QikProp tool embedded in Maestro was employed to assess the key pharmacokinetic parameters that play a fundamental role in determining the drug-like characteristics of a molecular entity. This goal is achieved through the comparison of the molecular properties against the criteria set by Lipinski’s rule of five. In accordance with Lipinski’s rule, a molecule is considered pharmaceutically viable if it adheres to the following criteria: a molecular weight not surpassing 500 Da, a count of hydrogen bond donors limited to five (donorHB ≤ 5), a restriction of hydrogen bond acceptors to not more than ten (acceptorHB ≤ 10), and a predicted octanol/water partition coefficient (QPlogPo/w) below 5 [59, 60]. In Table 2, it can be noted that all the hit molecules satisfied the rule of five criteria with no violation. Moreover, the solubility parameter (QPlogS) was within the accepted reference range (-6.5˗0.5) for the three hit compounds. Furthermore, two parameters associated with the potential for cellular membrane penetration underwent a thorough examination. The first is QPlogBB, which serves as an indicator of blood-brain barrier (BBB) permeability, and the second is QPPCaco-2, reflecting cell membrane permeability. It is noteworthy that all the hit compounds under investigation exhibited favorable characteristics in terms of good cellular permeability while concurrently demonstrating limited capacity to traverse the BBB. Besides, a crucial parameter, QPlog HERG, responsible for predicting IC50 values associated with the inhibition of HERG K+ channels, as outlined in Table 2, indicated that none of the compounds showed signs of cardiotoxicity. Collectively, the examination of all the pharmacokinetic parameters under study suggests that the candidate molecules hold promise as potential pharmaceutical agents.

**Table 2:** The pharmacokinetics parameters for the top three docked compounds on the NSD2 active pocket.

Hit	Mwt^a^	DHB^b^	AHB^c^	QPlogPo/w^d^	QPlogS^e^	QPlogHERG^f^	QPPCaco-2^g^	QPlogBB^h^	ROF^i^
**SN0450102**	312.278	5	5	0.303	-2.659	-5.159	16.169	-2.583	0
**SN0410255**	312.278	4	5	0.868	-3.279	-5.269	30.055	-2.212	0
**SN0142336**	257.245	3	3	1.315	-2.826	-4.825	174.487	-1.321	0
**Acceptable ranges**	>500	≤ 5	≤ 10	-2.0˗6.5	-6.5˗0.5	Below -5	>25 poor <500 great	-3˗1.2	0˗4

(a) Mwt (molecular weight). (b) DHB (hydrogen bond donor). (c) AHB (hydrogen bond acceptor). (d) QPlogPo/w (predicted partition coefficient in Octanol/Water). (e) QPlogS (predicted aqueous solubility). (f) QPlogHERG (Predicted IC50 for HERG K+ Blockade). (g) QPPCaco-2 (Predicted Caco-2 Cell Permeability). (h) QPlogBB (Predicted Brain/Blood partition coefficient). (i) ROF (Number of Lipinski’s Rule of Five Violations).

### Molecular dynamics (MD) simulation analysis

Molecular dynamics simulations evaluate the time-based stability of the simulated protein, considering the impact of bound ligands throughout the duration of the simulation. This approach aids in the refinement and validation of docking outcomes by assessing the dependability of predicted binding modes and discerning compounds that are unable to sustain stable interactions. By accounting for variables such as temperature and solvent effects, MD simulations offer valuable insights into the stability, flexibility, and conformational changes of the protein-ligand complexes. Herein, the complexes of the top three docked hits and the co-crystallized reference inhibitor were simulated for a duration of 200 nanoseconds on the Desmond package. The aim of the simulation was to further consolidate the matter of the suitability of the docked hits as inhibitors of the NSD2 pathway and by default assess their reliability as new anticancer candidates. For this purpose, the trajectories of MD were taken for extensive analysis by virtue of the essential statistical parameters.

**Fig 6:**
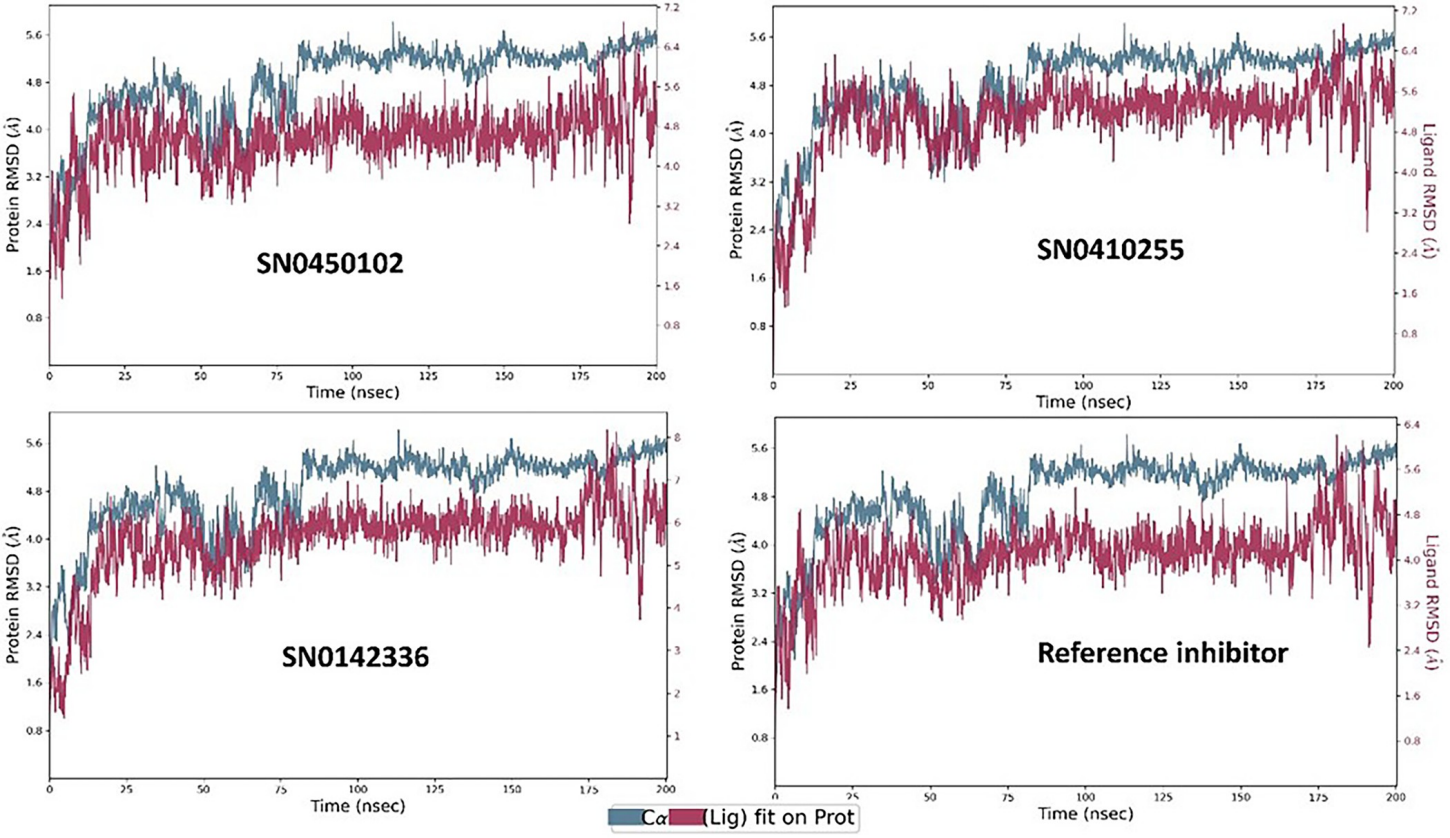
The RMSD plots of the simulated top three inhibitors and the co-crystallized reference inhibitor on NSD2 active site domain (PDB ID: 6G2O) for 200 nanoseconds.

**Fig 7:**
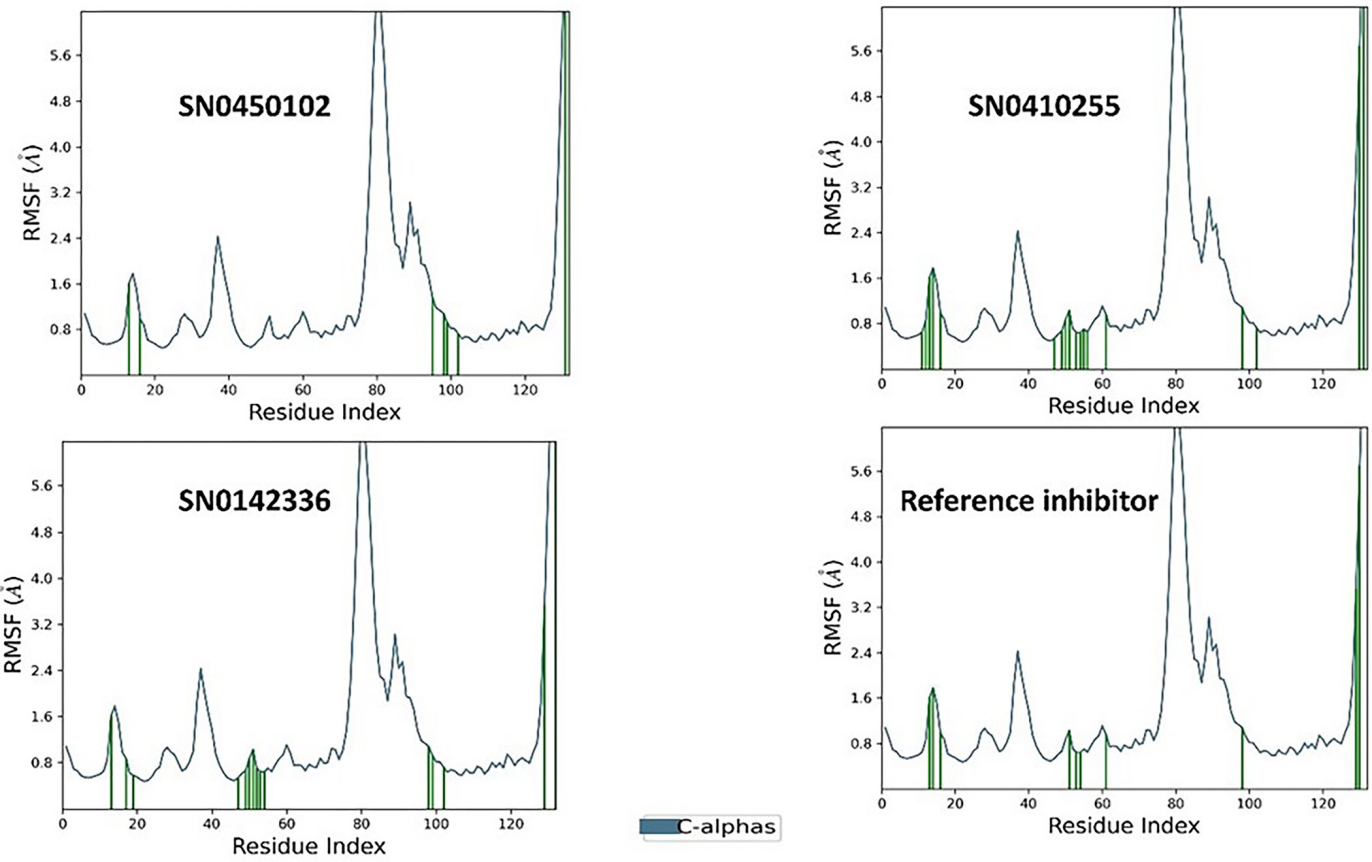
The protein RMSF plots of the simulated NSD2 active site domain (PDB ID: 6G2O) bound to the top three inhibitors and the co-crystallized reference inhibitor on for 200 nanoseconds.

**Fig 8:**
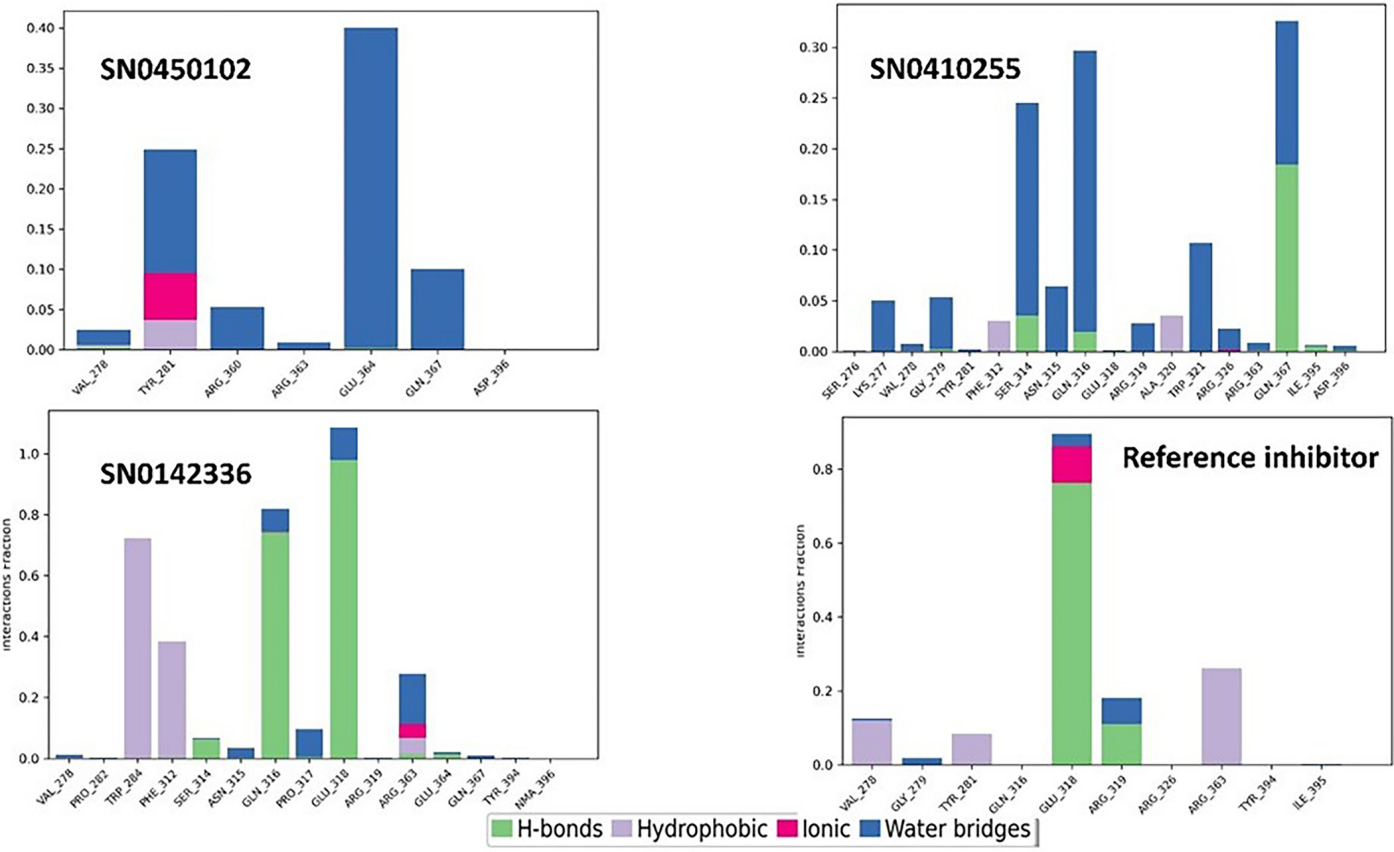
The protein-ligand contact histograms for the simulated top three inhibitors and the co-crystallized reference inhibitor on NSD2 active site domain (PDB ID: 6G2O) for 200 nanoseconds.

Importantly, root mean square deviation (RMSD) stands as a crucial metric, employed to evaluate the stability and integrity of the protein’s structure. Upon binding to compounds at its active site, a protein may experience structural alterations that impact its conformational stability. RMSD fundamentally quantifies the square root of the average deviations from the mean distances within the complex formed by the protein and ligand. The examination of molecular dynamics trajectories, coupled with the analysis of RMSD plots, offers an insightful preliminary assessment of the stability of the simulated protein-ligand complexes as previously highlighted [61]. In the case of a globular protein, ensuring stability within a simulated system generally involves RMSD fluctuations within a span of 1 to 3 Å. Notably, as long as the RMSD value attains a consistent level of fluctuation, it can be reasonably considered indicative of a converged system [61]. The co-crystallized reference inhibitor presented a relatively stable plot of fluctuations throughout the duration of the simulation as illustrated in Fig 6. Comparatively, for the hit molecules and the reference under study, the plots of RMSD throughout the 200 ns seemed to be equilibrated after 80 ns. They showed similar fluctuation patterns with an average protein RMSD of 4.81 Å. The hits SN0450102, SN0410255, SN0142336 and the reference displayed ligand RMSD values of 4.54, 5.06, 5.60, and 4.08 Å, respectively. SN0450102 and SN0410255 exhibited comparable fluctuations with the reference. This strongly suggests that they possess an equivalent binding pattern and stability. Another crucial metric for the evaluation of the flexibility of proteins during molecular dynamics simulations is the root mean square fluctuation (RMSF). This property offers valuable insights into the variations observed in specific amino acid residues, facilitating an assessment of how ligand binding influences the overall stability of the protein structure [62]. For the hit molecules in this study, the average RMSF value of the co-crystallized reference inhibitor and the hits, SN0450102, SN0410255, and SN0142336, was 1.37 Å. In general, as observed in Fig 6, the patterns observed in root mean square fluctuation indicate that the simulated protein-ligand complexes exhibit a degree of stability, characterized by minimal fluctuations in the crucial amino acid residues as in Fig 7. The protein-ligand contact histogram in Fig 8 indicates the types of different interactions that were formed throughout the simulation time. Taken as a reference, the co-crystallized inhibitor demonstrated hydrogen bonds to GLU-318 (76%) and ARG-319 (11%) alongside a water bridge with ARG-319 7%. Hydrophobic contacts were also seen with VAL-278 (12%), and ARG-363 (26%).

As per the histogram of SN0450102, it is noticed to engage in water-mediated hydrogen bonds with multiple amino acids: TYR-281 (15%), ARG-360 (5%), GLU-364 (40%), and GLN-367 (10%). Additionally, it did form hydrophobic contacts and ionic interactions with TYR-281 for 4% of the simulation time. The hit SN0410255 formed direct hydrogen bonds with the key amino acids SER-314 (4%) and GLN-367 (19%). Moreover, it also formed an indirect water-bridged hydrogen bond for 5% of the simulation time with LYS-277, GLY-279, ASN-315, and ARG-319. Likewise, it presented a water bridge with SER-314 (21%) and GLN-316 (29%). The hit SN0142336 formed direct hydrogen bonds with GLN-316 (74%) and GLU-318 (98%) together with bridged hydrogen bonds with GLN-316, GLU-318, ARG-363, and PRO-317.

In comparing the whole contacts, the hits SN0450102, SN0410255, and SN0142336 showcased diverse binding mechanisms. Hit SN0450102 relies on water-mediated hydrogen bonds and hydrophobic contacts, while hit SN0410255 demonstrates specificity through direct and water-bridged hydrogen bonds. Hit SN0142336 emphasizes direct hydrogen bonds with a pronounced affinity for GLN-316 and GLU-318. The reference inhibitor shares similarities with the hit SN0142336 but displays a broader hydrophobic interaction profile. Understanding these binding modes is critical for designing targeted inhibitors with optimal therapeutic efficacy.

**Fig 9:**
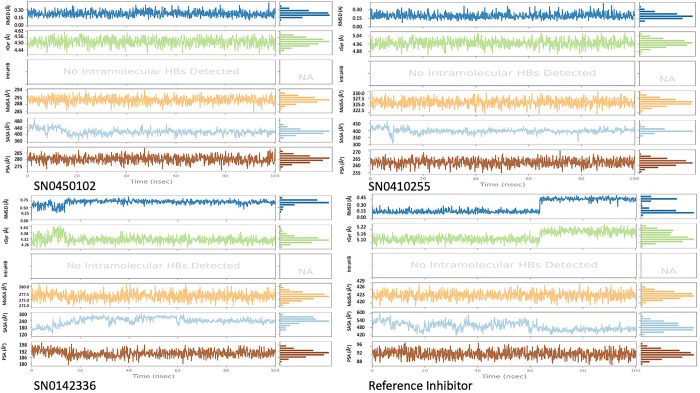
Ligand properties of the simulated top three inhibitors and the co-crystallized reference inhibitor on NSD2 active site domain (PDB ID: 6G2O) for 200 nanoseconds.

Several ligand properties were studied, including Ligand RMSD, rGyr, MolSA, and SASA, as depicted in Fig 9. The rGyr (radius of gyration) is considered a significant marker of protein compactness. The average rGyr values of SN0450102, SN0410255, SN0142336, and the reference were 4.52 ± 0.036, 4.97 ± 0.04, 4.31 ± 0.04, and 5.15 ± 0.04 Å, respectively. To study the protein’s exposure to solvent molecules and its structural stability, three surface areas were examined, including MolSA (molecular surface area), SASA (solvent-accessible surface area), and PSA (polar surface area). For SN0450102, the MolSA, SASA, and PSA average values were 289.87 ± 1.3199, 416.95 ± 18.63, 280.028 ± 2.47 Å^2^, respectively. In the case of SN0410255, the corresponding average values were 326.13 ± 1.24 Å^2^ for MolSA, 400.60 ± 18.08 Å^2^ for SASA, and 261.93 ± 2.30 Å^2^ for PSA. For SN0142336, the averages were 276.35 ± 1.33 Å^2^ for MolSA, 239.81 ± 28.64 Å^2^ for SASA, and 190.45 ± 2.95 Å^2^ for PSA. Finally, for the reference, the averages were 423.51 ± 1.61 Å^2^ for MolSA, 487.75 ± 35.35 Å^2^ for SASA, and 92.27 ± 1.86 Å^2^ for PSA.

It can be recapped from these observations that these three compounds offer unique opportunity for NSD2 repression since they tackle influential amino acid residues in the PWWP1 domain with outstanding binding affinities, interaction stability, and oral bioavailability. Moreover, being natural promises a good safety profile.

The binding affinity and binding mode of the top compounds with NDS2 can further be investigated experimentally to ascertain its biological fitness using differential static light scattering (DSLS), surface plasmon resonance (SPR) assay and reverse ITC titration. Ligand based drug design such as QSAR studies is a very convenient approach to accelerate the development of compounds by studying the large number of molecules that interact with the biological target of interest [63]. These studies can be done to further explore the structures activity relationship of these NSD2 inhibitors. The selectivity of these compounds toward other NDS isoforms (NDS1 and NDS3) which also linked to cancer can be explored for therapeutic development against cancer. Combinatorial studies of these potential NSD2 inhibitors with other therapies such as EZH2 inhibitors can be studied for effective cancer treatment.

## Conclusion

NSD2 mal-expression has been linked with cancer development and proliferation, therefore it gained tremendous attention as a drug target for cancer management, especially in cases of AML. This study explored SuperNatural 3.0 utilizing a multi-faceted approach that included pharmacophore modeling, molecular docking, and molecular dynamics simulations to identify potential inhibitors for NSD2. After rigorous computational analysis, three leads promised superior docking scores i.e., binding the reference inhibitor. Ligand-residue interaction analysis highlighted diverse binding mechanisms, while pharmacokinetic profiling indicated drug-like characteristics. Molecular dynamics simulations over 200 nanoseconds confirmed the stability of the observed interactions between NSD2 and the selected hits. Overall, these compounds show potential as anti-cancer agents targeting NSD2, Nonetheless, further experimental validation is warranted to affirm these conclusions.

## Supporting information

S1 File The online version contains supplementary material available at xxx. **S1 Fig**: The histogram shows the distribution of HTVS docking scores for the top 100 compounds selected for the XP molecular docking.; **S1 Table**: HTVS dockings scores for the top 100 compounds selected for the XP molecular docking.
(DOCX)
