# Supplementary material for: Unveiling the potential of phytochemicals to inhibit nuclear receptor binding SET domain protein 2 for cancer: Pharmacophore screening, molecular docking, ADME properties, and molecular dynamics simulation investigations
Source: PLoS One. 2024 Aug 20;19(8):e0308913. doi: 10.1371/journal.pone.0308913 (PMC11335128; doi:10.1371/journal.pone.0308913)
Supplement: S1 File — S1 Fig: The histogram shows the distribution of HTVS docking scores for the top 100 compounds selected for the XP molecular docking.; S1 Table: HTVS dockings scores for the top 100 compounds selected for the XP molecular docking. (DOCX) [file pone.0308913.s001.docx]

Supplementary Materials

Unveiling the Potential of Phytochemicals to Inhibit Nuclear Receptor Binding SET domain protein 2 for cancer: Pharmacophore screening, molecular docking, ADME properties, and molecular dynamics simulation investigations

Gamal A. Mohamed, ^1,*^ Hossam M. Abdallah ^1^, Ikhlas A. Sindi ^2^, Sabrin R. M. Ibrahim ^3,4^*, Abdulrahim A. Alzain ^5^

^1^ Department of Natural Products and Alternative Medicine, Faculty of Pharmacy, King Abdulaziz University, JeddahSaudi Arabia; hmafifi@kau.edu.sa (H.M.A.)

^2^ Department of Biology, Faculty of Science, King Abdulaziz University, Jeddah, Saudi Arabia; [easindi@kau.edu.sa](mailto:easindi@kau.edu.sa) (I.A.S)

^3^ Department of Chemistry, Preparatory Year Program, Batterjee Medical College, Jeddah, Saudi Arabia; sabrin.ibrahim@bmc.edu.sa

^4^ Department of Pharmacognosy, Faculty of Pharmacy, Assiut University, Assiut, Egypt

^5^Department of Pharmaceutical Chemistry, Faculty of Pharmacy, University of Gezira, Wad Madani, Sudan; [abdulrahim.altoam@gmail.com](mailto:abdulrahim.altoam@gmail.com) (A.A.A.)

***Corresponding author**

Email: [gahussein@kau.edu.sa](mailto:gahussein@kau.edu.sa) (G.A.M.); [sabrin.ibrahim@bmc.edu.sa](mailto:sabrin.ibrahim@bmc.edu.sa) (S.R.M.I.)


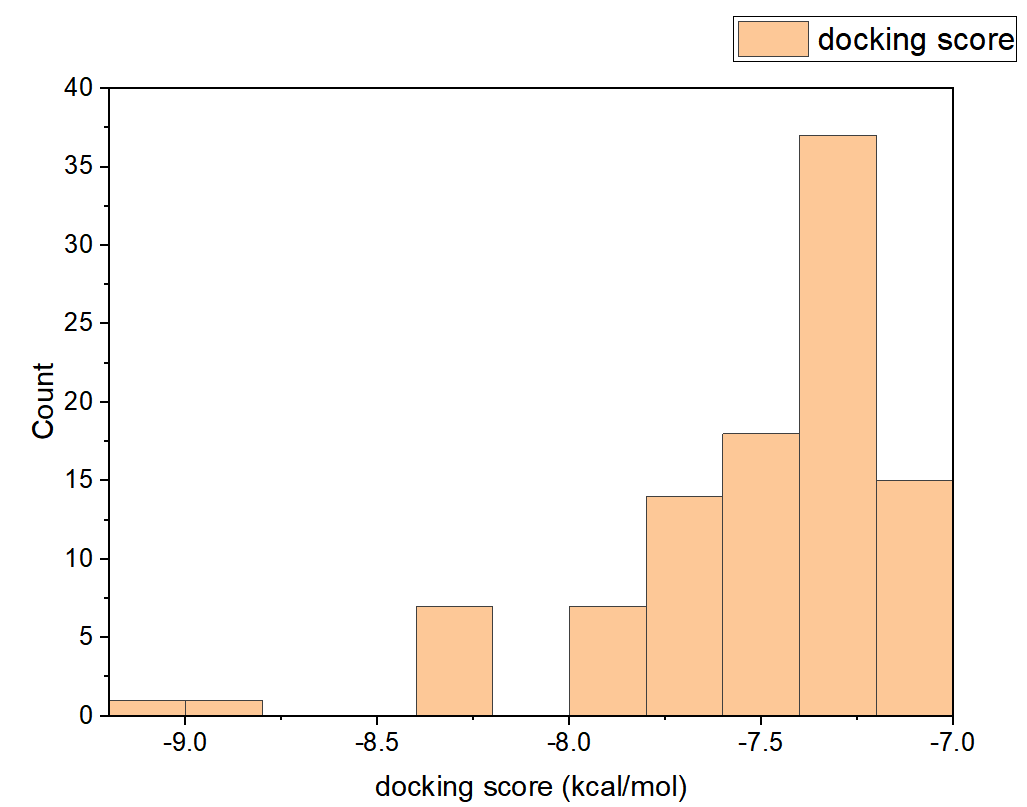


**Figure S1**: The histogram shows the distribution of HTVS dockings scores for the top 100 compounds selected for the XP molecular docking.

**Table S1**: HTVS dockings scores for the top 100 compounds selected for the XP molecular docking.

| Title | docking score |
| --- | --- |
| SN0142336 | -9.061 |
| SN0140921 | -8.965 |
| SN0156712 | -8.368 |
| SN0317452 | -8.364 |
| SN0474196 | -8.319 |
| SN0178237 | -8.298 |
| SN0232449 | -8.227 |
| SN0459442 | -8.212 |
| SN0038102 | -8.205 |
| SN0205852 | -8 |
| SN0241825 | -7.974 |
| SN0325155 | -7.961 |
| SN0306249 | -7.944 |
| SN0367681 | -7.851 |
| SN0396620 | -7.818 |
| SN0016799 | -7.801 |
| SN0456453 | -7.764 |
| SN0018915 | -7.726 |
| SN0261544 | -7.72 |
| SN0370058 | -7.717 |
| SN0321560 | -7.691 |
| SN0397015 | -7.689 |
| SN0166939 | -7.685 |
| SN0277250 | -7.67 |
| SN0389808 | -7.629 |
| SN0164638 | -7.626 |
| SN0352449 | -7.626 |
| SN0104839 | -7.624 |
| SN0123372 | -7.617 |
| SN0436937 | -7.605 |
| SN0470783 | -7.6 |
| SN0043489 | -7.575 |
| SN0254924 | -7.57 |
| SN0220002 | -7.564 |
| SN0431449 | -7.563 |
| SN0032046 | -7.551 |
| SN0450102 | -7.55 |
| SN0066808 | -7.539 |
| SN0410255 | -7.538 |
| SN0000879 | -7.526 |
| SN0238156 | -7.519 |
| SN0308866 | -7.458 |
| SN0339900 | -7.446 |
| SN0171229 | -7.438 |
| SN0317823 | -7.43 |
| SN0014050 | -7.425 |
| SN0460984 | -7.416 |
| SN0301389 | -7.406 |
| SN0154849 | -7.396 |
| SN0162311 | -7.392 |
| SN0475951 | -7.387 |
| SN0145295 | -7.375 |
| SN0457652 | -7.37 |
| SN0034009 | -7.369 |
| SN0073349 | -7.356 |
| SN0346214 | -7.354 |
| SN0114363 | -7.32 |
| SN0118574 | -7.32 |
| SN0234426 | -7.313 |
| SN0315059 | -7.311 |
| SN0156450 | -7.31 |
| SN0364857 | -7.31 |
| SN0256222 | -7.307 |
| SN0030463 | -7.307 |
| SN0040698 | -7.306 |
| SN0386980 | -7.305 |
| SN0303813 | -7.3 |
| SN0338657 | -7.297 |
| SN0091785 | -7.287 |
| SN0441675 | -7.284 |
| SN0123093 | -7.284 |
| SN0325639 | -7.281 |
| SN0407424 | -7.274 |
| SN0269612 | -7.27 |
| SN0025698 | -7.258 |
| SN0107573 | -7.247 |
| SN0139654 | -7.239 |
| SN0292225 | -7.239 |
| SN0323221 | -7.236 |
| SN0152913 | -7.232 |
| SN0348764 | -7.223 |
| SN0431360 | -7.221 |
| SN0472680 | -7.214 |
| SN0073391 | -7.206 |
| SN0336420 | -7.204 |
| SN0395559 | -7.2 |
| SN0348007 | -7.197 |
| SN0468772 | -7.195 |
| SN0189116 | -7.194 |
| SN0261769 | -7.19 |
| SN0318251 | -7.188 |
| SN0359395 | -7.185 |
| SN0179175 | -7.183 |
| SN0432732 | -7.177 |
| SN0390082 | -7.172 |
| SN0368885 | -7.172 |
| SN0329130 | -7.172 |
| SN0383088 | -7.171 |
| SN0057952 | -7.166 |
| SN0280972 | -7.164 |
